# 

**Published:** 1873-10

**Authors:** 


### CONTENTS:

### ORIGINAL COMMUNICATIONS.

ARTICLE I.—Proceedings of the Thirteenth Annual Meeting of the American Dental Association .......... 241

ARTICLE II.—Physiology of the Dental Structures. (Concluded.) By S. P. Cutler, D. D. S., M. D. .......... 266

ARTICLE III.—Honor to Whom Honor is Due. By W. F. Fundenberg, M. D. .......... 271

### SELECTED ARTICLES.

ARTICLE IV.—Dental Physiology. (Concluded.) By H. S. Chase, D. D. S., M. D. .......... 273

ARTICLE V.—The Growth and Reproduction of Bone. By V. S. Lindsley, M. D. .......... 280

### EDITORIAL, ETC.

Baltimore College of Dental Surgery .......... 284

American Academy of Dental Science .......... 284

Treatment of Teeth with Dead Pulps .......... 284

Replantation of Teeth .......... 286

### MONTHLY SUMMARY.

The Process of Taking Cold .......... 288

### OBITUARY.

Dr. Amos Westcott .......... 287

Dr. Joseph Robinson .......... 288

